# Can Mesenchymal Stem Cells Improve Bone Regeneration in Maxillary Sinus Augmentation? A Systematic Review and Meta‐Analysis

**DOI:** 10.1155/sci/6656563

**Published:** 2026-01-19

**Authors:** Franz Tito Coronel-Zubiate, Consuelo Marroquín-Soto, Sara Antonieta Luján-Valencia, Joan Manuel Meza-Málaga, Eduardo Luján-Urviola, Rubén Aguirre-Ipenza, Carlos Alberto Farje-Gallardo, Adriana Echevarría-Goche, Fredy Hugo Cruzado-Oliva, Heber Isac Arbildo-Vega

**Affiliations:** ^1^ Faculty of Health Sciences, Stomatology School, National University Toribio Rodriguez de Mendoza of Amazonas, Chachapoyas, 01001, Peru; ^2^ Department of Dentistry, School of Dentistry, Cientifica del Sur University, Lima, 15840, Peru; ^3^ Faculty of Dentistry, Dentistry School, Catholic University of Santa Maria, Arequipa, 04013, Peru; ^4^ Faculty of Dentistry, Andean University Nestor Caceres Velasquez, Juliaca, 21104, Peru; ^5^ Faculty of Health Sciences, Continental University, Lima, 15046, Peru; ^6^ Department of Dentistry, Dentistry School, Peruvian University Cayetano Heredia, Lima, 15046, Peru, upch.edu.pe; ^7^ Faculty of Stomatology, Stomatology School, National University of Trujillo, Trujillo, 13001, Peru, unitru.edu.pe; ^8^ Faculty of Dentistry, Dentistry School, San Martin de Porres University, Chiclayo, 14012, Peru, usmp.edu.pe; ^9^ Faculty of Human Medicine, Human Medicine School, San Martín de Porres University, Chiclayo, 14012, Peru; ^10^ Graduate School, National University Toribio Rodriguez de Mendoza of Amazonas, Chachapoyas, 01001, Peru

**Keywords:** bone regeneration, dental implants, maxillary sinus augmentation, mesenchymal stem cells, regenerative dentistry

## Abstract

**Background:**

Mesenchymal stem cells (MSCs) have shown promise in preclinical models for enhancing bone regeneration around dental implants. However, clinical evidence regarding their efficacy in maxillary sinus augmentation procedures for dental implants remains inconclusive.

**Objective:**

To evaluate the clinical effectiveness of MSC‐based regenerative therapies compared to conventional grafting in maxillary sinus augmentation for implant placement.

**Methods:**

A systematic review and meta‐analysis were conducted following Preferred Reporting Items for Systematic Reviews and Meta‐Analyses (PRISMA) 2020 guidelines and registered in PROSPERO (CRD42023488758). Electronic and gray literature searches were performed across six databases. Eligible studies included randomized clinical trials evaluating MSC‐based bone regeneration in maxillary sinus lifts. Risk of bias (RoB) was assessed using the Cochrane RoB 2.0 tool, and certainty of evidence was rated using the Grading of Recommendations Assessment, Development, and Evaluation (GRADE) methodology. Meta‐analyses were performed for implant success rate and bone formation outcomes.

**Results:**

Six randomized controlled trials (RCTs) were included, comprising 74 patients and 222 implants. Meta‐analysis revealed no statistically significant difference in implant success rate between MSC and control groups (risk ratio [RR] = 0.98; 95% confidence interval [CI]: 0.94–1.03; *p* = 0.50; *I* ^2^ = 11.69%). For bone neoformation, continuous data favored the control group (standardized mean difference [SMD] = –0.83; 95% CI: –1.37 to –0.30; *p* = 0.002; *I* ^2^ = 0%; indicating a medium to large effect size that represents a clinically perceptible advantage in bone formation for the control interventions), while dichotomous outcomes showed no significant difference (RR = 1.11; 95% CI: 0.73–1.67; *p* = 0.62). Sensitivity analyses confirmed the robustness of findings. The certainty of evidence was rated as high for bone formation outcomes and moderate for implant success.

**Conclusions:**

MSC‐based regenerative therapies do not appear to offer a significant clinical advantage over conventional grafting techniques in maxillary sinus augmentation. These results should be interpreted with caution, given the limited number of trials and clinical heterogeneity. Further well‐designed studies are needed to validate their efficacy in implant‐related bone regeneration.

## 1. Introduction

Peri‐implant bone loss remains one of the major clinical limitations in oral implantology, compromising implant stability, facial esthetics, and masticatory function for millions of patients worldwide [1, 2]. This condition is exacerbated by advanced age, periodontal disease, and systemic comorbidities such as osteoporosis and diabetes [3, 4], imposing a substantial burden on healthcare systems [5] and significantly reducing patients’ quality of life [6]. Conventional bone regeneration techniques—including autologous, alloplastic, and xenogeneic grafts—currently represent the clinical standard. However, their application is limited by the scarcity of donor tissue, morbidity associated with graft harvesting, unpredictable integration outcomes, and elevated surgical costs [7, 8]. These limitations have driven the search for safer and more effective therapeutic alternatives.

Bone defects encountered in oral implantology often vary in etiology, size, and morphology, including defects due to tooth extraction, sinus pneumatization, periodontal disease, or trauma. Such defects can be classified as horizontal, vertical, or through and through, affecting both alveolar ridge height and width. Autografts (harvested from intraoral or extraoral sites), allografts, xenografts, and synthetic biomaterials, such as deproteinized bovine bone mineral (DBBM), *β*‐tricalcium phosphate (*β*‐TCP), hydroxyapatite, bioactive glass, and composite scaffolds, are widely used substitutes to restore bone volume and provide osteoconductive frameworks. However, each biomaterial presents limitations, such as slow resorption, poor vascularization in large defects, risk of disease transmission for xenografts, donor site morbidity for autografts, and lack of osteoinductive capacity in many synthetic substitutes [9]⁠. Recent clinical evidence comparing bovine‐derived xenografts with synthetic graft materials in lateral maxillary sinus augmentation has confirmed these material‐dependent differences in regenerative outcomes [10].

Among these, mesenchymal stem cells (MSCs) have emerged as a promising biotechnological intervention due to their capacity to differentiate into osteoblasts, modulate the local immune response, and secrete pro‐regenerative factors that support vascularized bone formation [11, 12]. Various MSC sources—such as bone marrow, dental pulp, adipose tissue, and peripheral blood—have been employed in clinical protocols to promote alveolar regeneration, yielding encouraging results in clinical trials [13, 14]. The combination of MSCs with osteoconductive scaffolds, whether synthetic or biological, has been shown to enhance the quality of regenerated bone tissue and reduce osseointegration time [15]. However, despite the solid biological rationale and robust preclinical data, clinical evidence remains fragmented and methodologically inconsistent. Recent advancements in nano‐engineered delivery systems have demonstrated enhanced MSC viability, homing, and osteogenic performance, further supporting their translational potential [16].

MSCs can be derived from multiple tissue sources, including bone marrow, adipose tissue, dental pulp, periodontal ligament, and perinatal tissues. These sources differ in cell yield, proliferation capacity, osteogenic potential, immunomodulatory profile, and ease of harvesting. Furthermore, the behavior of MSCs in vivo is heavily influenced by the local microenvironment: vascular supply, oxygenation, host inflammatory status, and scaffold architecture all affect osteogenic differentiation and bone formation [17]. Clinical translation additionally faces challenges such as standardized processing of MSCs (isolation, expansion, and characterization), regulatory considerations, scaffold design (porosity, biodegradability, and mechanical stability), dosage, timing of cell delivery, and long‐term stability of regenerated bone. While preclinical animal models generally show favorable results combining MSCs with appropriate biomaterials, human studies remain limited and heterogeneous in methodology and outcome reporting [18].

Previous systematic reviews have attempted to address this topic, but present notable limitations. Some have excluded relevant cell sources such as MSCs derived from dental pulp or peripheral blood; others rely solely on narrative synthesis without quantitative analysis or employ incomplete search strategies and heterogeneous inclusion criteria [19–21]. Moreover, the use of evidence certainty assessment tools such as the Grading of Recommendations Assessment, Development, and Evaluation (GRADE) is scarce, and summary of findings tables—essential for clinical interpretation—are rarely reported. Subgroup analyses based on cell source, application protocol, or bone defect characteristics are often lacking, as are stratified meta‐analyses or sensitivity analyses. These gaps hinder a comprehensive understanding of the differential effects of these emerging therapies.

To address these limitations, the present systematic review and meta‐analysis of clinical trials evaluates the clinical efficacy of MSCs in bone regeneration for oral implantology. This review incorporates multiple MSC sources, clinically relevant outcomes such as bone neoformation rate and healing time, and includes an analysis of risk of bias (RoB), heterogeneity, and certainty of evidence. The findings aim to inform clinical decision‐making, update therapeutic guidelines, and support the regulatory development of regenerative therapies in oral implantology.

## 2. Methods

### 2.1. Study Design and Eligibility Criteria

A systematic review and meta‐analysis were conducted in accordance with the Preferred Reporting Items for Systematic Reviews and Meta‐Analyses (PRISMA) 2020 guidelines. The complete PRISMA checklist is available in Supporting Information 1 (PRISMA 2020 checklist). The review protocol was registered in the International Prospective Register of Systematic Reviews (PROSPERO) under the identification code CRD42023488758.

The research question was formulated using the PICO framework:

P (population): Adults presenting with posterior maxillary atrophy requiring maxillary sinus augmentation as a preparatory procedure for dental implant placement.
I (intervention): Treatment with MSCs for bone regeneration.
C (comparator): Bone regeneration procedures without stem cells (e.g., platelet concentrates, autologous grafts, or biomaterials).
O (outcomes): Bone neoformation rate or percentage, bone quality, and healing time.

Eligible studies were randomized or nonrandomized clinical trials assessing the clinical efficacy of MSCs in bone regeneration procedures related to oral implantology. Studies were required to report detailed information regarding the bone marrow aspiration site, cell processing methods, regeneration protocols, and clinical or histological outcomes. No restrictions were applied regarding language or publication date. Studies were excluded if they were systematic reviews, in vitro or animal studies, case series, cohort or case–control studies, duplicate publications, or unpublished data.

Although nonrandomized trials were eligible during the screening phase, only randomized controlled trials (RCTs) met all inclusion criteria and were included in the final synthesis. This choice ensured higher methodological rigor and allowed for the use of the Cochrane RoB 2.0 tool in RoB assessment.

The decision to include exclusively RCTs in the final synthesis was based on their superior ability to minimize bias and confounding, providing a more reliable estimation of treatment effects. While nonrandomized studies can offer valuable insights, integrating them with RCTs in meta‐analyses may introduce inconsistency or bias if not properly adjusted. The GRADE approach emphasizes the separation or cautious integration of evidence from randomized and nonrandomized studies to maintain methodological rigor and transparency [22].

### 2.2. Information Sources and Search Strategy

An electronic search was conducted in the following databases: PubMed/MEDLINE, Cochrane Library, EMBASE, Scopus, Web of Science (WoS), and ScienceDirect, from inception to June 2024 without language or date restrictions. In addition, gray literature sources were explored by screening OpenGrey and the first 100 results of Google Scholar, ordered by relevance. This restriction was applied to ensure feasibility and consistency, as Google Scholar does not provide reproducible search filters or sorting options. Non‐peer‐reviewed content and duplicates were excluded during the screening phase. A manual search was also performed in specialized journals in implant dentistry, and the reference lists of included studies were screened for additional eligible articles.

Search strategies were developed using Medical Subject Headings (MeSH) and relevant keywords, combined with the Boolean operators “AND” and “OR.” Terms included: “dental implants,” “stem cells,” “mesenchymal stem cells,” “bone regeneration,” and “osteoconduction,” among others. The detailed search strategies for each database are available in Supporting Information 2 (database‐specific search strategies).

### 2.3. Study Selection and Data Collection

The selection process was performed independently by two reviewers (Franz Tito Coronel‐Zubiate and Carlos Alberto Farje Gallardo) in two phases: an initial screening of titles and abstracts, followed by a full‐text review of potentially eligible studies. Discrepancies were resolved through discussion, or when necessary, by consultation with a the third reviewer (Rubén Aguirre‐Ipenza). The Rayyan QCRI web tool (Rayyan Systems Inc., https://www.rayyan.ai/) was used to manage and streamline the screening process.

Finally, regarding the study design, although the initial protocol considered the possibility of including nonrandomized studies, only RCTs met the final eligibility criteria. This approach ensured higher methodological rigor, minimized potential biases, and allowed the application of a standardized RoB assessment using the Cochrane RoB 2.0 tool. Consequently, the evidence synthesis reflects a homogeneous methodological standard while maintaining transparency in the selection process.

Data extraction was also performed independently by two reviewers (Consuelo Marroquín‐Soto and Fredy Hugo Cruzado‐Oliva) using a predesigned Excel spreadsheet. Discrepancies during data extraction were resolved by the third reviewer (Franz Tito Coronel‐Zubiate). The extracted data included: first author, year of publication, country, study design, number of participants, mean age, number of implants, type of bone regeneration procedure, stem cell harvesting and processing methods, donor and recipient sites, graft materials, follow‐up duration, timing of implant placement, and implant success rate. Quantitative outcomes related to bone neoformation and other clinically relevant parameters were also extracted.

The review collected data on methodological, clinical, and outcome‐related characteristics of the included studies. General variables included the first author, year of publication, country of origin, study design, sample size, mean age, number of implants, type of regenerative procedure, MSC source and processing method, donor and recipient sites, type of biomaterial used, follow‐up duration, and implant placement modality. The primary outcomes were bone neoformation, reported as volume or percentage, in millimeters, cubic millimeters, or percentage depending on the study, quality of the regenerated bone, assessed by histological analysis, presence of residual biomaterial, and parameters such as bone volume fraction (BVF), and healing or bone formation time, measured in months from the regenerative procedure to biopsy or implant placement. These outcomes were selected based on their clinical relevance in implant dentistry and their availability in the included studies. A secondary outcome was the implant success rate following bone regeneration, which was recorded when reported.

To avoid ambiguity in the interpretation of results, we clearly defined outcome prioritization. The primary outcome was bone neoformation (volume or percentage), based on clinical and histomorphometric relevance. Secondary outcomes included healing time and bone quality descriptors (such as BVF), while implant success rate was treated as an exploratory outcome when reported, and was not included in the primary quantitative synthesis. This hierarchy allowed consistent synthesis and interpretation of results across heterogeneous studies.

Although nonrandomized trials were eligible during the screening phase, only RCTs met all inclusion criteria and were included in the final synthesis. This choice ensured higher methodological rigor and allowed for the use of the Cochrane RoB 2.0 tool in RoB assessment.

During the eligibility phase, 32 full‐text articles were assessed. Of these, 26 [23–48] were excluded for not meeting the inclusion criteria. The specific reasons for exclusion are detailed in Supporting Information 3: Table S1.

### 2.4. RoB Assessment

The RoB in the included clinical trials was independently assessed by two reviewers (Sara Antonieta Luján‐Valencia and Joan Manuel Meza‐Málaga) using the RoB 2.0 tool developed by the Cochrane Collaboration. Any disagreements were resolved through consensus.

**Figure 1:**
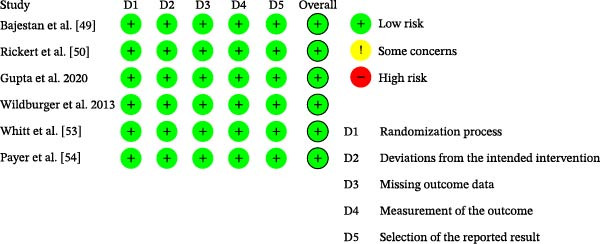
Risk of bias summary (RoB 2.0).

Six studies were evaluated [49–54], and all were rated as having a low overall RoB, which supports the internal validity of the synthesized findings. A summary of RoB assessment is presented in Figure 1, and the full judgments are detailed in Supporting Information 4 (RoB 2.0 summary matrix).

As previously detailed in Section 2, the six included studies were assessed for methodological quality using the RoB 2.0 tool from the Cochrane Collaboration. All trials were judged to present a low overall RoB, supporting the internal validity of the data synthesis. The summary of RoB assessments is presented in Figure 1 (RoB 2.0 summary figure), and detailed in Supporting Information 4 (RoB 2.0 full judgments for included studies).

### 2.5. Data Synthesis and Sensitivity Analysis

Meta‐analyses were conducted using random‐effects or fixed‐effects models depending on the level of heterogeneity (*I* ^2^). Statistical analyses and sensitivity testing were performed by Heber Isac Arbildo‐Vega using appropriate software. For each pooled estimate, sensitivity analysis was performed using the leave‐one‐out method to assess the influence of individual studies on the overall effect size. This approach involved recalculating the meta‐analysis iteratively by removing one study at a time. The robustness of the results was confirmed when effect estimates remained consistent across all iterations. Sensitivity plots are presented in Supporting Information 5: Figures S1 and S2.

The choice between fixed‐effect and random‐effects models was guided by the degree of heterogeneity across studies, evaluated using the *I* ^2^ statistic. Random‐effects models were preferred in the presence of moderate‐to‐high heterogeneity, as they account for both within‐study and between‐study variance, providing more conservative estimates. Conversely, fixed‐effect models were used when heterogeneity was negligible, assuming a single true effect across studies. This methodological decision is consistent with current best practices in meta‐analytical modeling and ensures accurate interpretation of pooled results [55, 56].

The certainty of evidence was evaluated using the GRADE framework. This approach considers five domains: RoB, inconsistency, indirectness, imprecision, and publication bias. In this review, all domains were carefully assessed for each outcome. The inclusion of only RCTs allowed a higher initial certainty level, which was downgraded only when substantial concerns were identified in any domain [22].

## 3. Results

### 3.1. Study Selection

**Figure 2:**
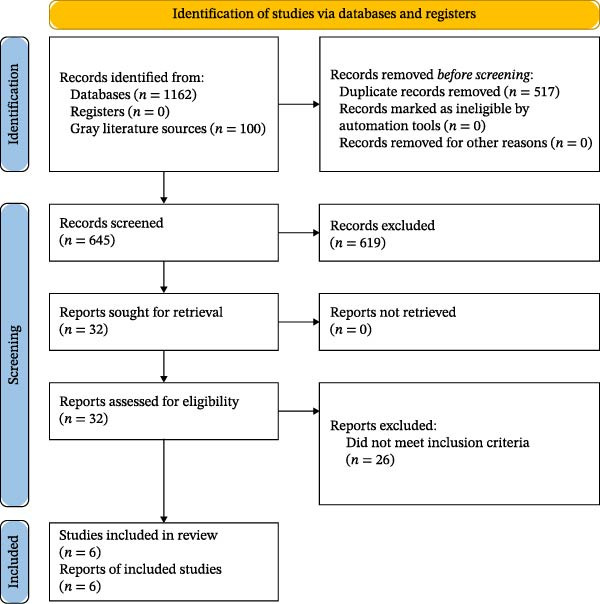
PRISMA 2020 flow diagram of study selection.

The initial search across PubMed/MEDLINE, Scopus, WoS, Cochrane Library, and ScienceDirect yielded a total of 1162 records. After the removal of 517 duplicates, 645 titles and abstracts were screened. Of these, 32 full‐text articles were assessed for eligibility, and six studies met the inclusion criteria and were included in this systematic review [49–54]. No additional studies were identified through manual reference screening or supplementary sources. The selection process is illustrated in Figure 2 (PRISMA 2020 flow diagram).

### 3.2. Characteristics of the Included Studies

Six RCTs were included, encompassing a total of 74 patients and 222 placed implants. Sample sizes ranged from 6 to 20 participants, with bilateral sinus lift procedures commonly used as the regenerative model in four studies [50, 52–54]. These four trials adopted a split‐mouth randomized controlled design, in which bilateral sinus lift sites within the same patient served as test and control sides. The mean age of participants ranged from 27 to 60.8 years; three studies involved populations with a mean age over 55 years [50, 52, 54], while one study focused on a younger cohort [51].

Regarding interventions, four trials utilized autologous bone marrow aspiration from the iliac crest or proximal tibia as the source of MSCs [49–51, 54], while two studies employed commercially prepared cellular allografts (Osteocel Plus) [51, 53]. In the autologous MSC studies, the cells were combined with biomaterials such as DBBM or *β*‐TCP. In contrast, the allografts included preseeded stem cells within the graft matrix. Donor sites included the iliac crest [50, 54] and proximal tibia [49, 52], with aspirated volumes ranging from 10 to 60 mL. Gupta et al. [51] included both graft types in comparative study arms. The recipient site in all studies was the atrophic maxillary sinus, justifying the use of sinus lift procedures as the basis for bone regeneration prior to implant placement. Implants were placed in a delayed manner, typically 3–6 months postregeneration, with clinical follow‐up ranging from 4 to 12 months [50, 53].

### 3.3. Synthesis of Results

#### 3.3.1. Meta‐Analysis of Implant Success Rate

**Figure 3:**
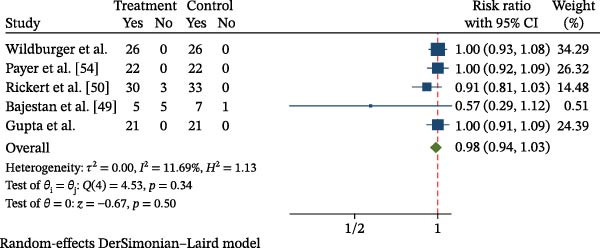
Forest plot of implant success rate (random‐effects meta‐analysis).

A total of five studies [49–52, 54] were included in the meta‐analysis evaluating implant success rate following bone regeneration procedures using MSCs. A random‐effects model (DerSimonian–Laird) was applied, yielding a pooled risk ratio (RR) of 0.98 (95% confidence interval [CI]: 0.94–1.03; *p* = 0.50), indicating no statistically significant difference between the MSC and control groups. Heterogeneity was low (*I* ^2^ = 11.69%; *τ* ^2^ = 0.00; *Q* = 4.53, *p* = 0.34), suggesting overall consistency across studies (Figure 3). Across the included trials, reported implant success rates were generally high. Three studies [51, 52, 54] achieved 100% success, while Rickert et al. [50] reported a slightly lower rate of 91% in the MSC group. Bajestan et al. [49] observed a success rate of 50% in the MSC group versus 85.7% in controls. Whitt et al. [53] also reported complete implant survival. Despite this variability, leave‐one‐out sensitivity analysis confirmed the robustness of the pooled estimate (Supporting Information 5: Figure S1), showing no substantial change in direction or statistical significance upon sequential exclusion of each study.

#### 3.3.2. Meta‐Analysis of Bone Regeneration

**Figure 4:**
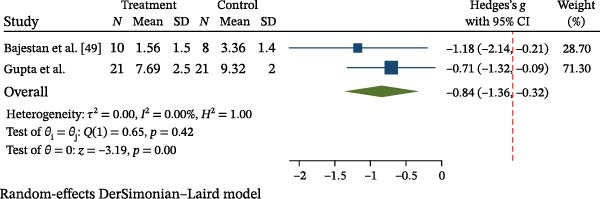
Forest plot of bone regeneration (MSC vs. control—continuous outcome).

**Figure 5:**
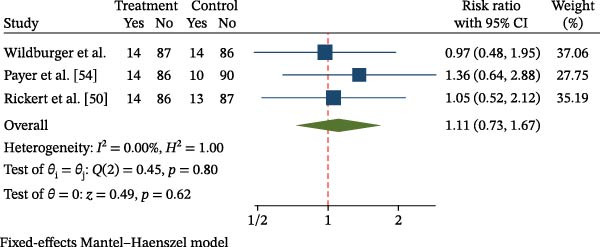
Forest plot of bone regeneration success (MSC vs. control—dichotomous outcome).

Two studies [49, 53] contributed data to a meta‐analysis comparing the extent of bone regeneration between MSC‐based therapies and conventional grafting approaches. Using a random‐effects model and standardized mean difference (SMD) as the effect measure, the pooled SMD was –0.83 (95% CI: –1.37 to –0.30), indicating a statistically significant difference favoring the control group (*p* = 0.002). No heterogeneity was detected (*I* ^2^ = 0%; *p* = 0.44) (Figure 4). Three studies [50, 52, 54] valuated new bone formation as a dichotomous outcome (presence or absence of bone regeneration). A fixed‐effects Mantel–Haenszel model was applied, yielding a pooled RR of 1.11 (95% CI: 0.73–1.67; *p* = 0.62), indicating no statistically significant difference between MSC‐treated and control groups. No heterogeneity was detected across studies (*I* ^2^ = 0%; *Q* = 0.45, *p* = 0.80) (Figure 5). Leave‐one‐out sensitivity analysis confirmed the robustness of the findings, as no substantial change in effect size or statistical significance was observed when omitting any individual study (Supporting Information 5: Figure S2).

### 3.4. Descriptive Synthesis of Bone Formation Outcomes

Additional studies provided quantitative and qualitative assessments of bone formation. Wildburger et al. [52] and Payer et al. [54] reported new bone formation rates of 13.5% and 14.17%, respectively. Rickert et al. [50] documented a vertical bone gain of 0.47 mm, while Bajestan et al. [49] observed a mean gain of 2.9 ± 1.3 mm. Whitt et al. [53] described bone formation in both anterior and posterior sinus regions, though no numerical values were reported. These results are summarized in Table 1.

**Table 1:** Summary of bone regeneration outcomes in the included clinical trials.

Author(s)	*n*	Age	Study design	Number of implants	Follow‐up (months)	Success rate	Bone regeneration	Donor site	Recipient site	Treatment
Wildburger et al. [52]	7	58 (47–72)	RCT, split‐mouth	52	3–6	100%	13.5% vs. 13.9%; no significant difference	Iliac crest	Maxillary sinus (bilateral)	MSC + DBBM (BioOss) vs. DBBM only
Payer et al. [54]	6	58.2 (43–70)	RCT, split‐mouth	44	3–6	100%	14.17% vs. 10.41%–5.25%; no significant difference	Tibia	Maxillary sinus	MSC + DBBM (BioOss) vs. DBBM only
Rickert et al. [50]	12	60.8 (48–69)	RCT, split‐mouth	66	4–12	91% (MSC) vs. 100%	0.47 mm vs. 0.41 mm; no significant difference	Iliac crest	Maxillary sinus	MSC + DBBM (BioOss) vs. autograft + DBBM
Bajestan et al. [49]	18	T:27 (18–42) C:31 (19–54)	Controlled clinical trial	T:10 C:8	4	50% (MSC) vs. 87.5%	2.9 ± 1.3 mm (MSC) vs. 3.6 ± 1.6 mm (control)	Iliac crest/mandibular ramus	Alveolar ridge	MSC + *β*‐TCP vs. autograft block + allogeneic particulate bone
Whitt et al. [53]	11	>22	Controlled pilot trial	NR	4	100%	Anterior: 12.35 mm (44.1%) and posterior: 17.27 mm (50.1%) between both groups	Cellular allograft	Maxillary sinus	Osteocel Plus (MSC allograft) vs. AlloOss (particulate cortico‐cancellous allograft)
Gupta et al. [51]	20	36.7 (18–65)	RCT, split‐mouth	42	6	100%	7.69 ± 2.5 mm (MSC) vs. 9.32 ± 2.0 mm (control)	Peripheral blood	Maxillary sinus	Peripheral MSCs vs. blood clot

### 3.5. Certainty of Evidence (GRADE)

The certainty of evidence was assessed using the GRADE approach across the main outcomes: implant success rate, bone neoformation (continuous), and bone formation presence (yes/no). A Summary of Findings table was constructed in GRADEpro GDT and is provided in Supporting Information 6 (GRADE evidence profile).

For the outcome “bone regeneration (continuous)," the evidence was rated as high certainty. The included RCTs had low RoB, consistent results, and narrow CI (SMD: –0.84; 95% CI: –1.36 to –0.32; outcome: bone regeneration), with no concerns for indirectness or imprecision.

For the “presence or absence of bone formation” (evaluated as a dichotomous outcome), the certainty of evidence was also rated as high, based on data from three RCTs. No serious concerns were identified in any of the GRADE domains.

In contrast, the certainty of evidence for the “implant success rate” outcome was rated as moderate, due to concerns regarding inconsistency among studies. While most trials reported 100% implant survival, one study showed a markedly lower success rate in the MSC group, introducing variability in the pooled estimate. No important concerns were identified regarding RoB, indirectness, or publication bias.

## 4. Discussion

### 4.1. Summary of Main Results

This systematic review and meta‐analysis evaluated the clinical efficacy of MSCs in bone regeneration for implant therapy, specifically within the context of maxillary sinus augmentation [10]. Overall, MSC‐based therapies did not demonstrate a statistically significant improvement in implant success rates when compared to conventional grafting approaches. However, in terms of bone neoformation, modest but inconsistent differences were observed, with some trials paradoxically favoring control groups. These findings must be interpreted in light of the limited sample sizes, variability in cell sources and scaffolds, and the evolving understanding of MSCs in peri‐implant bone metabolism [3, 11, 12].

### 4.2. Overall Completeness and Applicability of Evidence

The included RCTs focused exclusively on sinus lift procedures in systemically healthy adults. Although this regenerative model is clinically relevant and widely used, it does not fully represent the diversity of indications, anatomical sites, and patient profiles in which MSCs might be applied. Importantly, patients with systemic comorbidities such as diabetes—a well‐established risk factor for peri‐implant disease—were excluded from all included studies [4]. Moreover, outcomes related to patient‐centered variables such as quality of life or long‐term implant stability were not assessed, limiting broader clinical applicability. Emerging trials using MSCs in atrophic mandibular ridges and anterior maxilla have shown promising regenerative results outside the sinus context, expanding their therapeutic scope [57].

### 4.3. Certainty of the Evidence

Although all included studies were RCTs and judged to have low RoB, the overall certainty of the evidence was downgraded due to imprecision, indirectness, and inconsistency in outcome definitions and measurement tools. Imprecision was particularly evident in the bone neoformation outcomes, often based on small sample sizes and differing histomorphometric protocols.

Some pooled estimates, particularly for bone neoformation, were based on only two to three studies. This limited number reduces the statistical power of the meta‐analysis and introduces instability in the effect estimates. As such, while meta‐analytic techniques were applied, these results should be interpreted with caution. The certainty of evidence was downgraded accordingly in the GRADE assessment, reflecting both imprecision and inconsistency across studies.

Nonetheless, the underlying biological rationale for using MSCs in bone regeneration remains strong, given their osteoinductive and immunomodulatory capacities, which have been demonstrated in both preclinical and translational research [7, 12, 13, 16]. Furthermore, recent mechanistic studies highlight the role of MSCs in modulating local inflammation, supporting angiogenesis, and accelerating early stages of bone healing [16, 58]. These effects have been confirmed in animal models and are now being validated in ongoing human clinical trials [59].

In addition to the RCTs included, recent clinical and translational studies provide deeper insight into the practicality and limitations of using MSCs in maxillary sinus augmentation. For instance, the study “bone regeneration using mesenchymal stromal cells and scaffolds (2023)” [60] reviewed nine clinical trials involving MSC‑scaffold constructs, highlighting that MSCs from bone marrow, combined with bioceramic scaffolds, often improve bone defect repair, although the degree of improvement varies and adverse events are rare. Another study by Ivanovski et al. [61], “The therapeutic use of dental mesenchymal stem cells (2024)” emphasizes the importance of standardization in MSC source, dosage, and scaffold type for consistent outcomes in oromaxillofacial regeneration contexts. These findings reinforce that while MSC‐based therapies show biological promise (e.g., angiogenesis, immunomodulation, and enhanced osteogenesis), their clinical efficacy in sinus lift remains inconsistent due to variability in protocol, scaffold biocompatibility, and patient‐specific factors.

It is worth noting that, although the eligibility criteria permitted the inclusion of both randomized and nonrandomized clinical trials, only RCTs met all inclusion parameters. This decision minimized the potential for selection and performance bias and enhanced the internal validity of the synthesized findings. By focusing exclusively on RCTs, this review provides a more reliable estimation of treatment effects, although at the cost of reduced generalizability to real‐world clinical settings, where such trial conditions may not always be replicable.

In addition to imprecision and indirectness, substantial clinical heterogeneity was observed across studies. This included variation in donor sites (e.g., iliac crest and tibia), grafting materials (e.g., DBBM, *β*‐TCP, and autografts), and cell processing methods. Such heterogeneity limited direct comparability and contributed to the downgrading of evidence certainty.

Notably, some outcomes in the GRADE summary table were rated as “High” certainty despite being supported by only two or three trials with small sample sizes and high heterogeneity. This rating may overestimate the confidence in these findings. For example, the outcome of bone neoformation, although rated as high certainty, was based on limited data and showed wide variability in effect size. Future updates should apply stricter thresholds for upgrading evidence quality and include explicit justification for high GRADE ratings when statistical power and consistency are limited.

### 4.4. Agreements and Disagreements With Other Reviews

The present findings are largely consistent with previous systematic reviews, such as that by Egido‐Moreno et al. [18], which also reported mixed outcomes for MSC‐based bone regeneration in implantology. However, the present study differs in several key aspects; it was included only RCTs, applied GRADE methodology, and focused exclusively on sinus augmentation as a reproducible model of vertical bone regeneration. Additionally, this review integrates updated evidence up to early 2025, reflecting newer trends in scaffold selection and MSC preparation.

Fu et al. [62]conducted a preclinical meta‐analysis evaluating systemic MSC applications and reported improvements in bone mineral density and new bone formation compared to controls, supporting the biological rationale of stem cell use despite differences in administration routes and clinical translation. Similarly, Namjoynik et al. [63] demonstrated that human dental pulp stem cells (DPSCs) combined with scaffolds significantly enhanced bone regeneration in animal models when compared to scaffolds alone. While both reviews affirm the osteogenic potential of MSCs, they rely on preclinical models, in contrast to the clinical evidence synthesized in this review. This is consistent with the preclinical meta‐analysis by Moeenzade et al. [64], which demonstrated significantly enhanced bone regeneration using DPSCs, with large effect sizes for both bone area (SMD = 2.40) and volume (SMD = 1.85). These findings underscore the regenerative capacity of MSCs in controlled animal models, but also highlight the translational gap between experimental and clinical settings. This translational gap may be explained by several factors, including the biological differences between human and animal microenvironments, such as vascularization patterns and immune responses, that can influence stem cell survival and osteogenic potential. In addition, the standardization and scalability of cell dosing in clinical scenarios remain more challenging than in controlled laboratory settings, which could partly account for the lower magnitude of regenerative outcomes observed in human trials.

Conversely, a clinical systematic review and meta‐analysis by Niño‐Sandoval et al. [65] focused on sinus floor elevation with MSCs and reported no significant difference in implant survival or vertical bone gain when compared to conventional grafting. These results mirror the present findings, reinforcing the need for further trials evaluating clinical endpoints in humans.

Moreover, Theodosaki et al. [66] systematically reviewed scaffold‐based delivery of MSCs in maxillofacial regeneration, highlighting consistent improvements in osteoconduction and integration, yet also calling for greater standardization of scaffold composition and MSC sourcing. These findings complement the clinical focus of the present review and reinforce the consensus that, while MSC‐based therapies are promising, further clinical trials with robust methodology are needed to clarify their advantages over traditional grafting approaches.

Broader systematic reviews, including applications in oral and maxillofacial surgery [31], confirm the safety profile and regenerative potential of MSCs, but emphasize the need for standardization and multicenter collaboration. Additionally, Hamada et al. [32] recently published a randomized controlled clinical trial comparing autogenous dentin chips with xenografts in immediate implant placement with thin buccal bone, reporting comparable esthetic outcomes and favorable tissue integration.

### 4.5. Limitations and Strengths

Limitations of this review include the narrow scope of intervention sites (limited to maxillary sinus lifts), and the small number of eligible trials, which restricted subgroup analyses. A major limitation of this review is the limited number of eligible clinical trials (*n* = 6), each with relatively small sample sizes—typically ranging from 6 to 20 participants per group. This greatly restricts the statistical power to detect clinically meaningful differences and limits the generalizability of the findings across broader patient populations and clinical settings. This limitation also influenced the certainty assessment within the GRADE framework, particularly affecting the domain of imprecision, since small sample sizes widened CIs and reduced the precision of effect estimates. Additionally, the reporting of outcomes was heterogeneous, and long‐term follow‐up data were scarce. On the other hand, this review strictly adhered to PRISMA 2020, was registered in PROSPERO, used comprehensive search strategies, including gray literature, and applied validated tools (RoB 2.0 and GRADE), to assess evidence quality and bias risk. The exclusive use of RCTs and clinically homogeneous populations enhances the internal validity of findings. However, greater diversity in anatomical sites and patient characteristics is needed in future studies to better reflect the clinical reality of implant dentistry [8, 59].

Another important limitation was the heterogeneity of interventions across trials. Differences in the type and source of MSCs, scaffold materials, donor sites, and preparation protocols introduced variability that weakened the internal consistency of results. This variability may partially explain the lack of clear superiority of MSC‐based treatments in the pooled analysis. Additionally, clinical heterogeneity could also be attributed to the use of distinct donor sites for MSC harvesting (e.g., iliac crest, tibia, and mandibular ramus), which may influence cell viability and osteogenic potential. Nonetheless, this variability reflects real‐world clinical practice and was accounted for in the random‐effects model applied in the meta‐analysis.

While the initial scope included the possibility of incorporating nonrandomized designs, the final review was restricted to RCTs to minimize potential biases and ensure methodological consistency. This choice enhanced the reliability of the evidence synthesis, although it also reduced the number of eligible studies.

Moreover, although several outcomes were reported in the included studies (e.g., bone volume, healing time, and implant success), we prioritized bone neoformation as the primary outcome, based on its direct relevance to evaluating the regenerative potential of MSC‐based therapies. Healing time and bone quality (e.g., BVF) were considered secondary outcomes, while implant success rate was treated as exploratory, due to inconsistency in its reporting and potential confounding by implant technique or loading protocols. We acknowledge that clearer delineation of this outcome hierarchy in earlier sections could improve interpretability and transparency.

For future research, we recommend multicenter RCTs with larger sample sizes to improve statistical power, especially for continuous and long‐term outcomes. Standardized protocols for MSC harvesting, processing, characterization (e.g., cell viability and phenotype), scaffold composition (type, porosity, and biodegradability), and delivery methods are critical. It is also essential to include patient‐centered outcomes such as bone functional loading, implant stability over time, and quality of life measures. Additionally, trials involving medically compromised populations (e.g., diabetes and osteoporosis) and evaluation of safety outcomes (immunogenicity and long‐term graft integration) will be necessary to translate MSC therapies into routine clinical practice.

## 5. Conclusions

According to the findings of the present systematic review and meta‐analysis, no significant differences were observed in implant success rates or bone formation outcomes when comparing MSC‐based regenerative procedures with conventional grafting in maxillary sinus augmentation. Although MSCs demonstrate promising biological potential for bone regeneration, these results should be interpreted with caution due to the limited number of available clinical trials, the methodological and clinical heterogeneity among studies, and the overall moderate certainty of the evidence. Therefore, no definitive clinical recommendations can be made regarding the superiority of MSC‐based therapies in implant‐related bone regeneration. The clinical benefit of MSCs remains unproven at this stage. Further RCTs with standardized protocols, adequate statistical power, and long‐term follow‐up are needed to confirm their efficacy and applicability in clinical practice.

## Author Contributions

Franz Tito Coronel‐Zubiate contributed to conceptualization, methodology, data curation, formal analysis, writing – original draft, supervision, and project administration. Consuelo Marroquín‐Soto contributed to data curation, validation, and writing – review and editing. Sara Antonieta Luján‐Valencia participated in investigation, resources, and writing – review and editing. Joan Manuel Meza‐Málaga contributed to software, data analysis, and visualization. Eduardo Luján‐Urviola participated in investigation, formal analysis, and writing – review and editing. Rubén Aguirre‐Ipenza contributed to methodology, risk‐of‐bias assessment, and validation. Carlos Alberto Farje‐Gallardo provided supervision, critical review, and conceptual input. Adriana Echevarría‐Goche performed literature search, study selection, and data extraction. Fredy Hugo Cruzado‐Oliva was responsible for meta‐analysis, statistical review, and draft revision. Heber Isac Arbildo‐Vega contributed to writing – review and editing, graphical representation, and final approval.

## Funding

This research did not receive any specific grant from funding agencies in the public, commercial, or not‐for‐profit sectors. The article processing charge (APC) will be covered by the Universidad Nacional Toribio Rodríguez de Mendoza de Amazonas (UNTRM), Peru.

## Conflicts of Interest

The authors declare no conflicts of interest.

## Supporting Information

Additional supporting information can be found online in the supporting information section.

## Supporting information


**Supporting Information 1** PRISMA 2020 checklist: Completed checklist of the PRISMA 2020 reporting guidelines used to structure this review.

**Supporting Information 2** Search strategies for all databases: Detailed electronic search strategies for PubMed/MEDLINE, Cochrane Library, EMBASE, Scopus, and Web of Science.

**Supporting Information 3** Table S1: List of excluded studies with reasons: Summary table listing all full‐text articles excluded after eligibility assessment and the reasons for their exclusion.

**Supporting Information 4** Risk of bias assessment: Visual summary and full breakdown of the risk of bias assessment using the Cochrane RoB 2.0 tool for all included trials.

**Supporting Information 5** Figures S1 and S2. Leave‐one‐out sensitivity analyses: Forest plots illustrating the robustness of the meta‐analytic findings for implant success rate and bone neoformation outcomes.

**Supporting Information 6** GRADE summary of findings table: Summary of the certainty of evidence for each primary outcome, prepared using GRADEpro GDT tool.

## Data Availability

All data generated or analyzed during this study are included in this published article and its supporting information. No additional data are available.
