# Supplementary material for: Can Mesenchymal Stem Cells Improve Bone Regeneration in Maxillary Sinus Augmentation? A Systematic Review and Meta‐Analysis
Source: Stem Cells Int. 2026 Jan 19;2026:6656563. doi: 10.1155/sci/6656563 (PMC12814210; doi:10.1155/sci/6656563)
Supplement: Supplementary file 2 — Supporting Information 2 Search strategies for all databases: Detailed electronic search strategies for PubMed/MEDLINE, Cochrane Library, EMBASE, Scopus, and Web of Science. [file SCI-2026-6656563-s002.docx]

**2. Supplementary Material 2 – Search Strategies for All Databases**

**PubMed/MEDLINE Search Strategy**

| **Database** | **PubMed** (<https://www.ncbi.nlm.nih.gov/pubmed/>)  **Date of Search:** June 2024 | | **Results** |
| --- | --- | --- | --- |
| **Search Strategy** | #1 | ("dental implants"[All Fields] OR "dental implant*"[All Fields] OR "mini implant*"[All Fields] OR "dental prosthes*"[All Fields]) AND ("stem cells"[All Fields] OR "stem cell*"[All Fields] OR "progenitor cell*"[All Fields] OR "mother cell*"[All Fields] OR "mesenchymal stem cell*"[All Fields]) AND ("bone regeneration*"[All Fields] OR "osteoconduction*"[All Fields]) | 239 |

**Cochrane Library Search Strategy**

| **Database** | **Cochrane Library** (<http://www.cochranelibrary.com/>)  **Date of Search:** June 2024 | | **Results** |
| --- | --- | --- | --- |
| **Search Strategy:** | #1 | MeSH descriptor: [Dental Implants] explode all trees | 4 |
|  | #2 | (dental implant*):ti,ab,kw |  |
|  | #3 | (mini implant*):ti,ab,kw |  |
|  | #4 | (dental prosthes*):ti,ab,kw |  |
|  | #5 | #1 OR #2 OR #3 OR #4 |  |
|  | #6 | MeSH descriptor: [Stem Cells] explode all trees |  |
|  | #7 | (stem cell*):ti,ab,kw |  |
|  | #8 | (progenitor cell*):ti,ab,kw |  |
|  | #9 | (mother cell*):ti,ab,kw |  |
|  | #10 | (mesenchymal stem cell*):ti,ab,kw |  |
|  | #11 | : #6 OR #7 OR #8 OR #9 OR #10 |  |
|  | #12 | MeSH descriptor: [Bone Regeneration] explode all trees |  |
|  | #13 | (Osteoconduction*):ti,ab,kw |  |
|  | #14 | #12 OR #13 #15: #5 AND #11 AND #14 |  |

**Web of Science Search Strategy**

| **Datebase** | **Web of Science** (<https://www.webofscience.com/>)  **Date of Search:** June 2024 | | **Results** |
| --- | --- | --- | --- |
| **Search Strategy** | #1 | (TS=( "dental implants" ) OR TS=( "dental implant*" ) OR TS=( "mini implant*" ) OR TS=( "dental prosthes*" )) AND ( TS=( "stem cells" ) OR TS=( "stem cell*" ) OR TS=( "progenitor cell*" ) OR TS=( "mother cell*" ) OR TS=( "mesenchymal stem cell*" )) AND (TS=( "Bone Regeneration*" ) OR TS=( "Osteoconduction*" )) | 289 |

**Embase Search Strategy**

| **Database** | **Embase** (<https://www.embase.com/>)  **Fecha de búsqueda:** Junio 2024 | | **Results** |
| --- | --- | --- | --- |
| **Search Strategy** | #1 | ('dental implants'/exp OR 'dental implants' OR 'dental implant*' OR 'mini implant*' OR 'dental prosthes*') AND ('stem cells'/exp OR 'stem cells' OR 'stem cell*' OR 'progenitor cell*' OR 'mother cell*' OR 'mesenchymal stem cell*') AND ('bone regeneration*' OR 'osteoconduction*') | 314 |

**Scopus Search Strategy**

| **Database** | **Scopus** (<https://www.scopus.com/>)  **Fecha de búsqueda:** Junio 2024 | | **Results** |
| --- | --- | --- | --- |
| **Search Strategy** | #1 | ( TITLE-ABS-KEY ( "dental implants" ) OR TITLE-ABS-KEY ( "dental implant*" ) OR TITLE-ABS-KEY ( "mini implant*" ) OR TITLE-ABS-KEY ( "dental prosthes*" ) ) AND ( TITLE-ABS-KEY ( "stem cells" ) OR TITLE-ABS-KEY ( "stem cell*" ) OR TITLE-ABS-KEY ( "progenitor cell*" ) OR TITLE-ABS-KEY ( "mother cell*" ) OR TITLE-ABS-KEY ( "mesenchymal stem cell*" ) ) AND ( TITLE-ABS-KEY ( "Bone Regeneration*" ) OR TITLE-ABS-KEY ( "Osteoconduction*" ) ) | 313 |
