# Supplementary material for: Can Mesenchymal Stem Cells Improve Bone Regeneration in Maxillary Sinus Augmentation? A Systematic Review and Meta‐Analysis
Source: Stem Cells Int. 2026 Jan 19;2026:6656563. doi: 10.1155/sci/6656563 (PMC12814210; doi:10.1155/sci/6656563)
Supplement: Supplementary file 3 — Supporting Information 3 Table S1: List of excluded studies with reasons: Summary table listing all full‐text articles excluded after eligibility assessment and the reasons for their exclusion. [file SCI-2026-6656563-s003.docx]

# Supplementary Table 1. Full-text Articles Excluded After Eligibility Assessment (n = 26)

| **Author** | **Year** | **Reason for Exclusion** |
| --- | --- | --- |
| Bulgin D, et al. | 2017 | Not a randomized or controlled clinical trial |
| Attia S, et al. | 2020 | Used platelet-rich plasma (not stem cell intervention) |
| Naik C, et al. | 2023 | Non-randomized trial, focused on membranes |
| Zheng RC, et al. | 2014 | In vitro / non-clinical model |
| Costa FH, et al. | 2023 | In vitro study – radiotherapy effects |
| Ito K, et al. | 2011 | Study design unclears or preclinical |
| Ito K, et al. | 2006 | Preclinical or mixed intervention study |
| Wang M, et al. | 2023 | Used allogeneic vs. autologous grafts, no MSCs |
| Tresguerres FGF, et al. | 2021 | Surgical technique study, no MSC intervention |
| Shoeib M, et al. | 2022 | Preprint, no peer-review; not MSC-focused |
| Solakoglu Ö, et al. | 2019 | Material comparison, no MSCs |
| Mohammed EEA, et al. | 2022 | In vitro nanomaterial study |
| Asahina I, et al. | 2021 | Observational follow-up, not a trial |
| Zakrzewski W, et al. | 2020 | Comparison of nanomaterials and stem cells |
| Yu T, et al. | 2017 | Surface chemistry – in vitro |
| Park JH, et al. | 2016 | Preclinical animal study |
| Zheng RC, et al. | 2015 | Duplicate or overlapping publication |
| Omori M, et al. | 2015 | In vitro / experimental model |
| Cruz ACC, et al. | 2015 | Preclinical study |
| Li J, et al. | 2014 | Scaffold testing |
| Ghaffari M, et al. | 2013 | Material study – no clinical outcome |
| Yamada Y, et al. | 2013 | Not eligible design |
| Zollino I, et al. | 2012 | In vitro stem cell study |
| Soltan M, et al. | 2010 | Technique case report, not controlled |
| Kim SH, et al. | 2009 | Pilot model, not human clinical trial |
| Ueda M, et al. | 2005 | Case report series, not a trial |
