# Supplementary material for: Can Mesenchymal Stem Cells Improve Bone Regeneration in Maxillary Sinus Augmentation? A Systematic Review and Meta‐Analysis
Source: Stem Cells Int. 2026 Jan 19;2026:6656563. doi: 10.1155/sci/6656563 (PMC12814210; doi:10.1155/sci/6656563)
Supplement: Supplementary file 4 — Supporting Information 4 Risk of bias assessment: Visual summary and full breakdown of the risk of bias assessment using the Cochrane RoB 2.0 tool for all included trials. [file SCI-2026-6656563-s004.docx]

| **Study** | **Randomization process** | **Deviations from intended interventions** | **Missing outcome data** | **Measurement of the outcome** | **Selection of the reported result** | **Overall bias** |
| --- | --- | --- | --- | --- | --- | --- |
| Bajestan et al., 2017 | Low | Low | Low | Low | Low | Low |
| Rickert et al., 2014 | Low | Low | Low | Low | Low | Low |
| Wildburger et al., 2020 | Low | Low | Low | Low | Low | Low |
| Gupta et al., 2019 | Low | Low | Low | Low | Low | Low |
| Payer et al., 2014 | Low | Low | Low | Low | Low | Low |
| Whitt et al., 2020 | Low | Low | Low | Low | Low | Low |

Supplementary Material 3. Risk of Bias (RoB) 2.0 Assessment for Included Randomized Controlled Trials
