# Supplementary material for: Can Mesenchymal Stem Cells Improve Bone Regeneration in Maxillary Sinus Augmentation? A Systematic Review and Meta‐Analysis
Source: Stem Cells Int. 2026 Jan 19;2026:6656563. doi: 10.1155/sci/6656563 (PMC12814210; doi:10.1155/sci/6656563)
Supplement: Supplementary file 5 — Supporting Information 5 Figures S1 and S2. Leave‐one‐out sensitivity analyses: Forest plots illustrating the robustness of the meta‐analytic findings for implant success rate and bone neoformation outcomes. [file SCI-2026-6656563-s005.docx]

Supplementary Figure 1. Leave-One-Out Sensitivity Analysis for Implant Success Rate


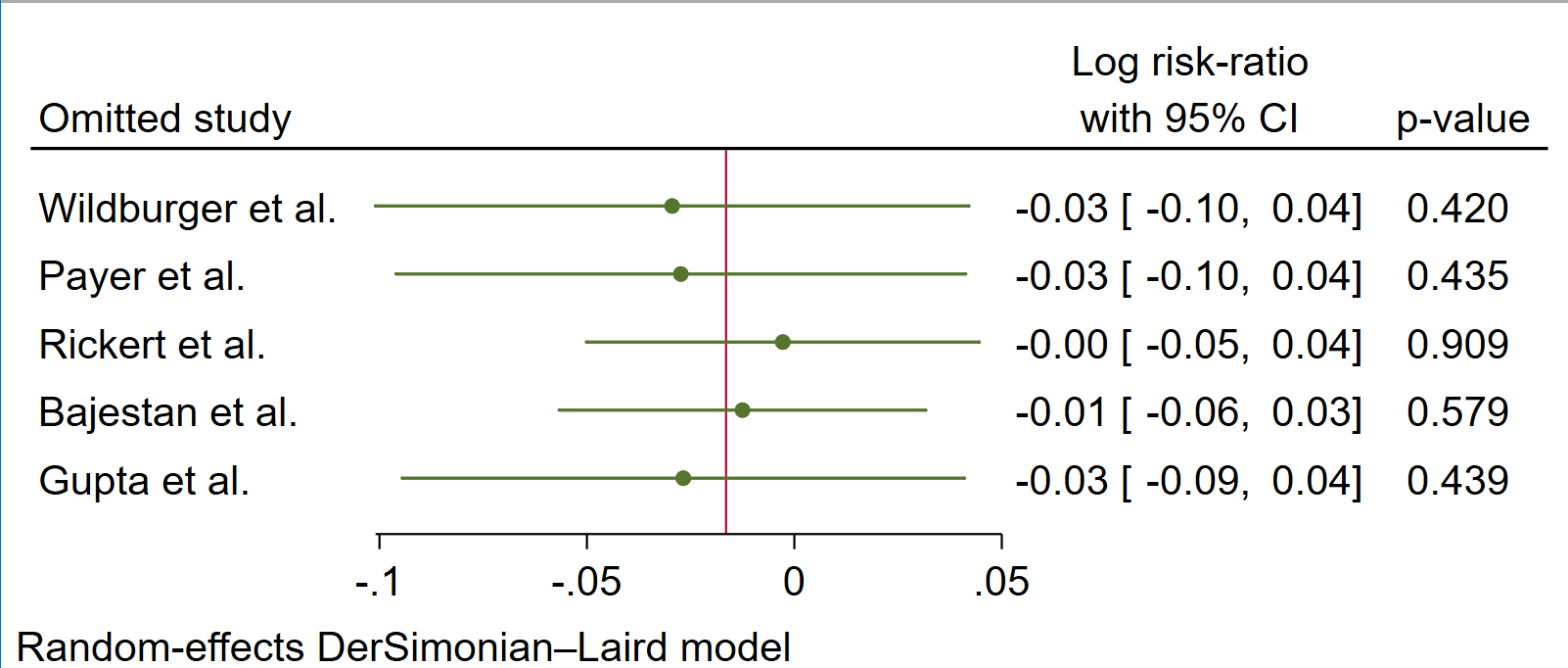


Supplementary Figure 2
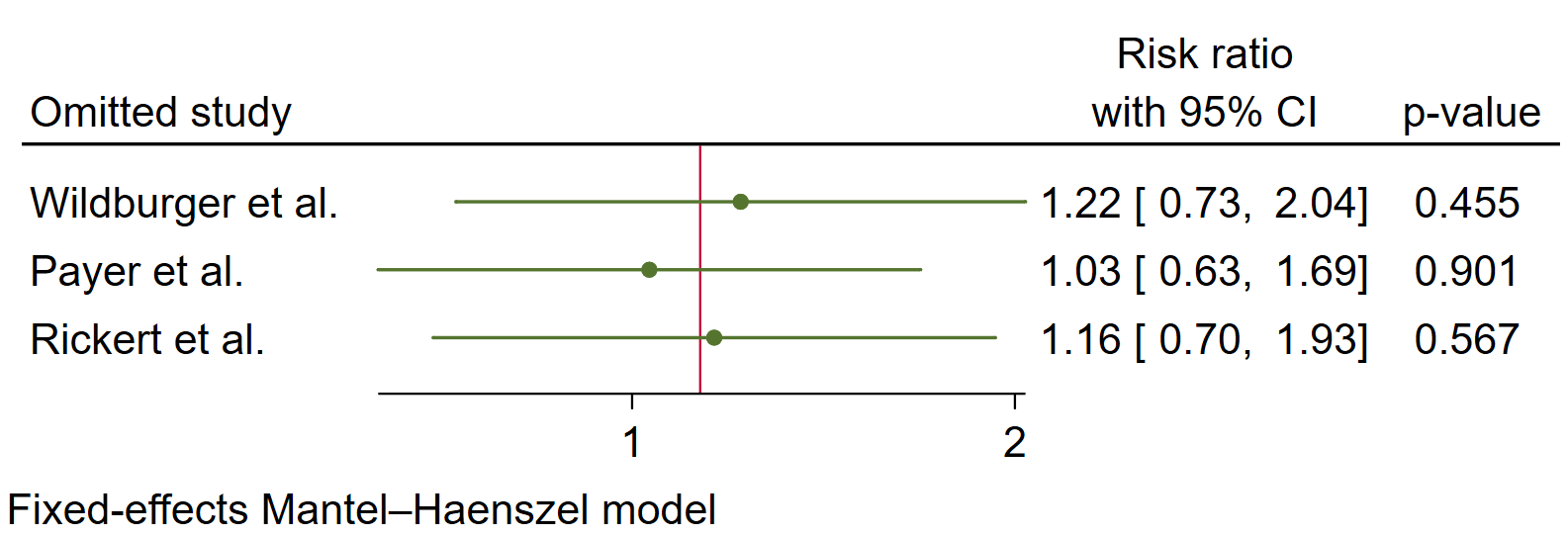
. Leave-One-Out Sensitivity Analysis for Bone Regeneration (Binary Outcome)
