# Supplementary material for: Can Mesenchymal Stem Cells Improve Bone Regeneration in Maxillary Sinus Augmentation? A Systematic Review and Meta‐Analysis
Source: Stem Cells Int. 2026 Jan 19;2026:6656563. doi: 10.1155/sci/6656563 (PMC12814210; doi:10.1155/sci/6656563)
Supplement: Supplementary file 6 — Supporting Information 6 GRADE summary of findings table: Summary of the certainty of evidence for each primary outcome, prepared using GRADEpro GDT tool. [file SCI-2026-6656563-s006.docx]

Supplementary Material 4 – Summary of Findings Table According to GRADE

| **Evaluación de certeza** | | | | | | | **№ patients** | | **Efect** | | **Certainty** | **Importancia** |
| --- | --- | --- | --- | --- | --- | --- | --- | --- | --- | --- | --- | --- |
| **No. of Studies** | **Study Design** | **Risk of Bias** | **Inconsistency** | **Indirectness** | **Imprecision** | **Other Considerations** | **MSC Group (n/N)** | **Control Group (n/N)** | **Relative Effect (95% CI)** | **Absolute Effect (95% CI)** |  |  |
| **Percentage of bone regeneration** | | | | | | | | | | | | |
| 3 | Randomized Trials | Not serious | Not serious | Not serious | Not serious | none | 42/162 (25.9%) | 37/162 (22.8%) | Not estimable | - | ⨁⨁⨁⨁ Alta |  |
| **Bone regeneration** | | | | | | | | | | | | |
| 2 | Randomized Trials | Not serious | Not serious | Not serious | Not serious | none | 31 | 29 | - | SMD **0.84 SD menor**  (1.36 to 0.32 lower) | ⨁⨁⨁⨁ Alta |  |
| **Success rate of implants** | | | | | | | | | | | | |
| 5 | Randomized Trials | Not serious | Serious | Not serious | Not serious | none | 104/260 (40.0%) | 109/260 (41.9%) | Not estimable |  | ⨁⨁⨁◯ Moderado |  |

**CI = Confidence Interval; SMD = Standardized Mean Difference**

***Note:*** *Although the number of studies is limited, the rating of "High" certainty was maintained due to consistency of effects, directness of evidence, and lack of serious imprecision (narrow confidence intervals).*
